# Population genetic study of 17 Y-STR Loci of the Sorani Kurds in the Province of Sulaymaniyah, Iraq

**DOI:** 10.1186/s12864-022-09005-6

**Published:** 2022-11-21

**Authors:** Balnd M. Albarzinji, Farhad M. Abdulkarim, Shaho A. Hussein, Dlshad Rashid, Hayder Lazim

**Affiliations:** 1 Kurdistan Institution for Strategic Studies and Scientific Research (KISSR), Sulaymaniyah, Iraq; 2 Microgene Diagnostic Center, Sulaymaniyah, Iraq; 3 College of Education, University of Sulaimani, Sulaymaniyah, Iraq; 4 School of Medicine, The Faculty of Health, Social Care and Medicine (FHSCM), Edge Hill University, Ormskirk, L39 4QP UK

**Keywords:** Population genetics, Y-STRs, Phylogenetic tree, Forensic genetics, Haplotype diversity, Y-haplogroup

## Abstract

**Background:**

The Kurds as an ethnic group are believed to be a combination of earlier Indo-European tribes who migrated and inhabited a mountainous area thousands of years ago. However, as it is difficult to describe the precise history of their origin, it is necessary to investigate their population relationship with other geographical and ethnic groups.

**Results:**

Seventeen Short Tandem Repeat markers on the Y chromosome (Y-STR) included in the AmpFLSTR™ Yfiler™ PCR Amplification Kit (Thermo Fisher Scientific, USA) were used to type DNA samples from the Sorani (Central) Kurdish population in Sulaymaniyah province. One hundred fifty-seven haplotypes were obtained from 162 unrelated male individuals. The highest and lowest gene diversities were DYS385a/b (GD = 0.848) and DYS392 (GD = 0.392), respectively. The haplotypes were used to predict the most likely haplogroups in the Sulaymaniyah population.

**Conclusion:**

Haplogroup prediction indicated predominance (28%) of subclade J2 (44/157) in the Sorani Kurds, northeast of Iraq. The pairwise genetic distance results showed that the Kurdish group clustered along with Asian populations, whereas the furthest countries were Europeans and Africans.

**Supplementary Information:**

The online version contains supplementary material available at 10.1186/s12864-022-09005-6.

## Background

The Kurds constitute the largest stateless nation in the world [1, 2]. Although no precise figures for the Kurdish population exist, a reasonable estimate is 30–35 million individuals [3–5]. Most of the Kurds inhabit a mountainous region straddling the borders of five countries: Iraq, Iran, Syria, Turkey and Armenia [4, 6]. Since Kurdish is a macro-language, the Kurds are also divided into at least five groups based on dialect: Kurmanji, Sorani, Kirmashani, Zazaki and Gorani [7].

The origin of the Kurds is uncertain [8, 9]. Traditionally, they are the descendants of different Indo-European tribes who migrated to the Zagros mountain region some 4000 years ago [5]. The earliest historical document of their existence in the region is the Sumerian cuneiform writing describing the land of Kurds (land of Karda), which dates to 3000 BC [10]. Nevertheless, other studies indicate that both the ethnic forefathers and the linguistic ancestors of the Kurds inhabited the Near East and Eurasia, geographically from outside and northwest of Iran, and they early occupied their Eurasian homeland [11].

Researchers are interested in using genetic information to extend knowledge about population histories and bio-geographic ancestry [12, 13]. Because the Y chromosome is inherited through the paternal line, Y-STRs are commonly used to understand migration history and the origin of populations worldwide [14–16]. Y-STRs are highly polymorphic with strong powers of discrimination among unrelated male individuals, making these markers suitable for human identification purposes. In forensic genetics, many Y-STR commercial kits have been developed as valuable tools for forensic analysis [16, 17]. Since Y-STRs have an average mutation rate of 0.2% per generation, they can also be used to study the genetic composition and consanguinity issues of various populations [18].

**Fig. 1:**
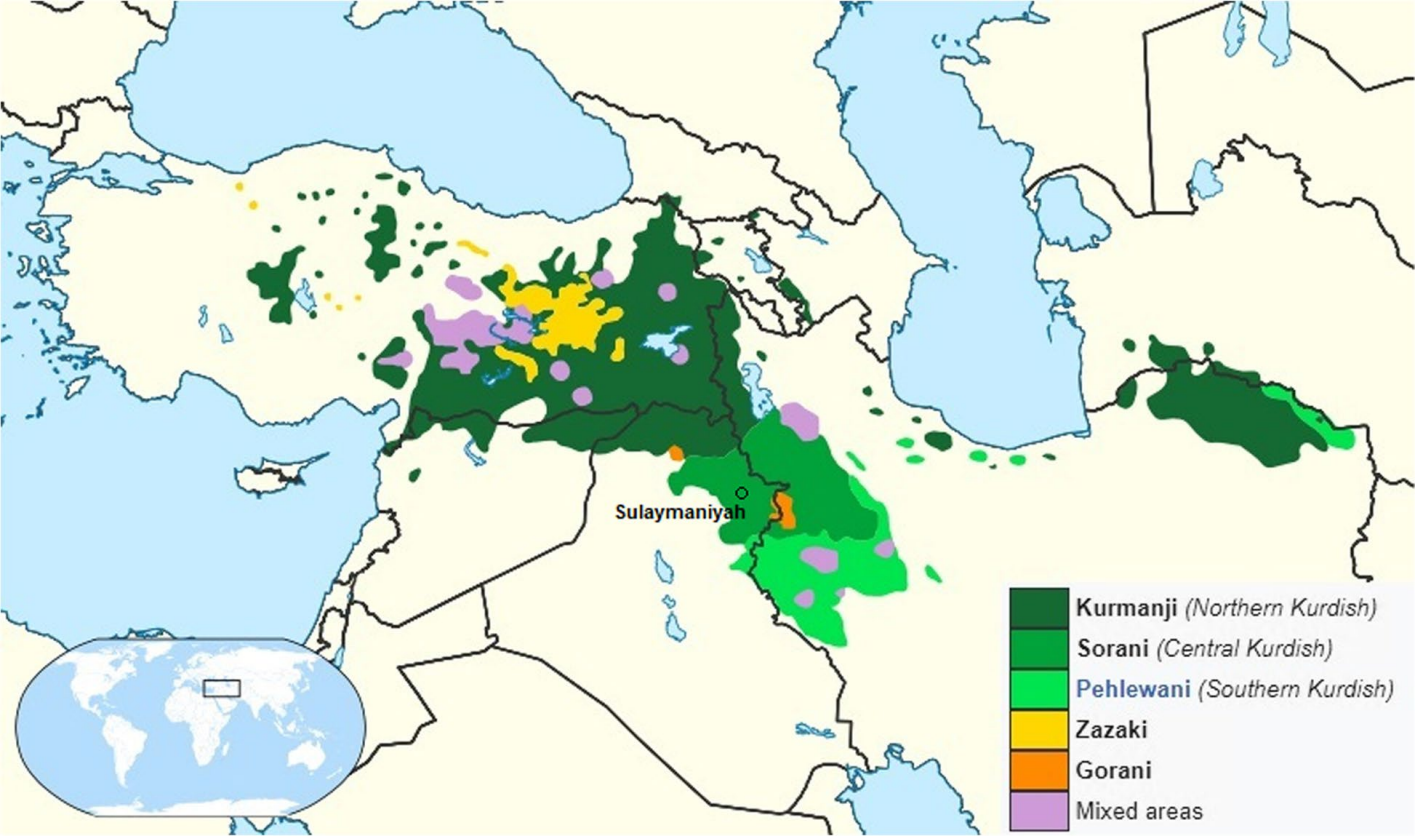
Map, adapted from Wikimedia Commons, showing the approximate distribution of the Kurds [21]

There is little genetic data available about the diversity of the Kurdish population. Furthermore, most previous studies collected DNA samples from Kurds as a single group for comparative analysis [19, 20]. Therefore, the current study focuses on the Sorani, Central Kurdish group, in the northeastern Iraqi province of Sulaymaniyah. It uses Yfiler (Thermo Fisher Scientific) with 17 loci to study the genetic variation of this population. The province of Sulaymaniyah is bordered by Iran in the east and by Iraqi Arabs in the south (Fig. 1) and has approximately 779,000 residents [22].

## Results

### Yfiler alleles diversity

**Fig. 2:**
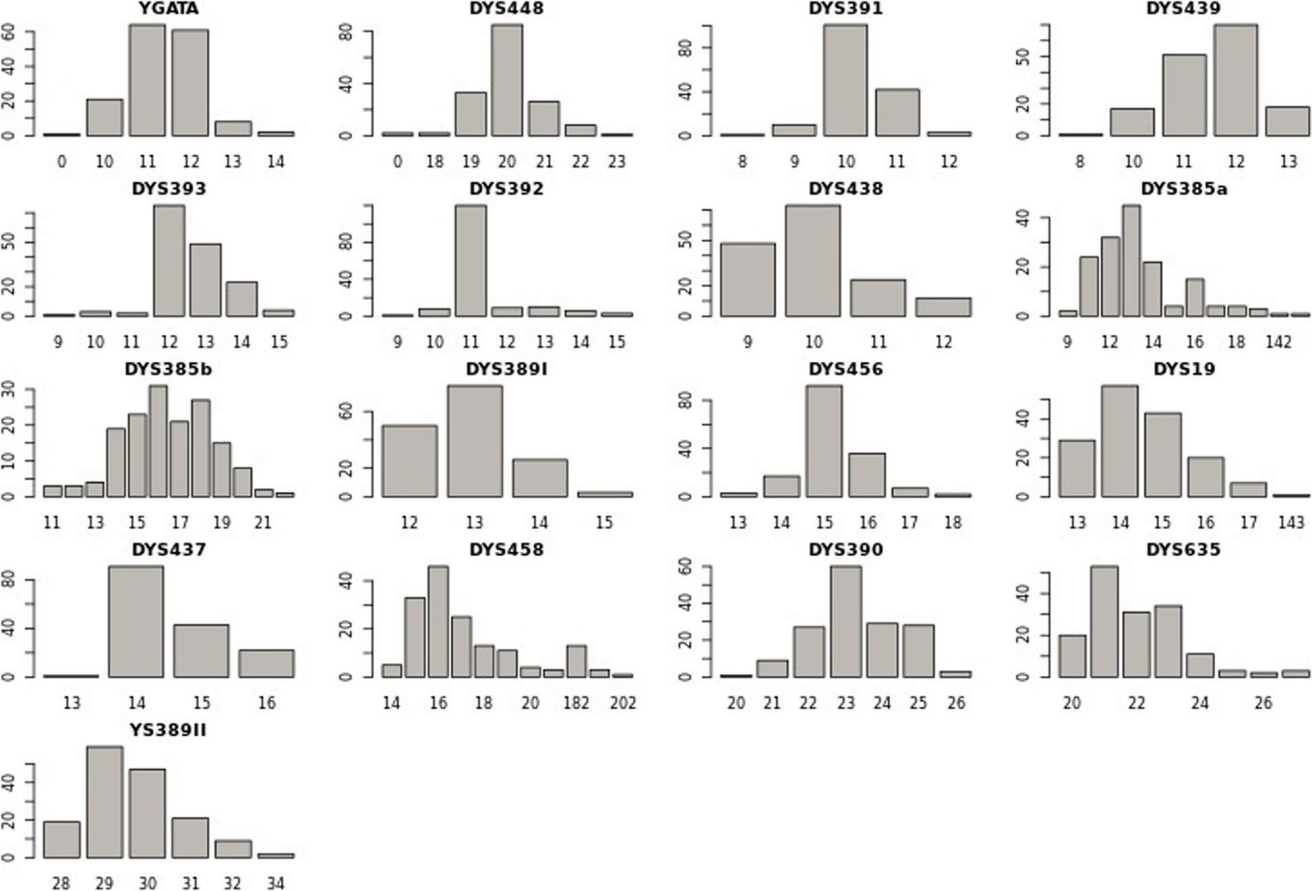
Distribution of allele frequencies of the Yfiler loci using the Sorani Kurd haplotypes

The majority of the haplotypes obtained in this study occur only once. A total number of 157 haplotypes were observed out of 162 samples; only five were shared among 5 individuals. Haplotype data is presented in Table S1, and has also been uploaded to the Y-chromosome STR haplotype reference database (YHRD), release R66 (accession number YA005683). Allele frequencies of the 17-STR loci were calculated using Genetic Analysis in Excel (GenAIEx 6.5) and presented in Table S2, while their distribution is shown in Fig. 2 below. The number of different alleles at each locus varied from 12 for DYS385a/b to 4 for DYS389I, DYS437 and DYS438. Gene diversity (GD), polymorphism information content (PIC), match probability (PM) and power of discrimination (PD) were calculated using the STR Analysis for Forensics (STRAF) online tool; the values for each locus are presented in Table S3. The results showed that the highest GD was at loci DYS385a/b and DYS458 (GD = 0.848 and 0.828 respectively) while the lowest was observed at locus DYS392 (GD = 0.392) (Fig. S1).

### Variant alleles

Several variant alleles were observed in different samples (Fig. S2). Duplicated alleles were found in one individual at locus DYS19 with values of 15, 16. Duplicated alleles were also found at locus YGATA H4 in one of the samples predicted to belong to haplogroup T-M184. In addition, a null-allele was found at two loci in three male individuals of the Sorani Kurdish population; DYS448 (two samples) were predicted to belong to haplogroups R1b-M343 and T-M184, and YGATA H4 (one sample) was predicted to belong to haplogroup J1-M267.

Microvariant alleles were observed in the Sorani Kurd males with a total percentage of 15.2% (24/157). Most were obtained at the locus DYS458, with 20 out of the 157 samples (12.7%); three samples were also observed at the locus DYS385a/b (1.9%) and one at the locus DYS19. Most of the microvariant samples (20/24) are predicted to belong to haplogroup J (J1 = 17 and J2 = 3).

Twelve samples (7.6%) were observed as mono-allele for the bi-allelic DYS385a/b locus. Several variant alleles were observed in our dataset that migrated outside the range of the allelic ladder of the AmpFLSTR™ Yfiler™ PCR Amplification Kit. The off-ladder alleles were at locus DYS635 allele 27 (three samples), predicted to belong to haplogroup R2-M124; and at locus Y GATA H4 allele 14 (two samples) predicted to belong to haplogroup E1b1b-M35.

### Haplogroup prediction and network analysis

Y-haplogroups were inferred by using Whit Athey’s Haplogroup Predictor tool (31-haplogroup-2021 version). The major sub-haplogroups of the Sorani Kurd were E1b1b-M35 (16.5%), J1-M267 (14%), J2a-M410 (12.7%), G2a-P15 (10.8%), J2a (8.9%), R1a-M17 (7.6%) and R1b-M343 (7%). The haplogroup distributions in the Sorani Kurdish population are shown in Fig. S3. The complete haplogroup predictions are shown in Table S4.

**Fig. 3:**
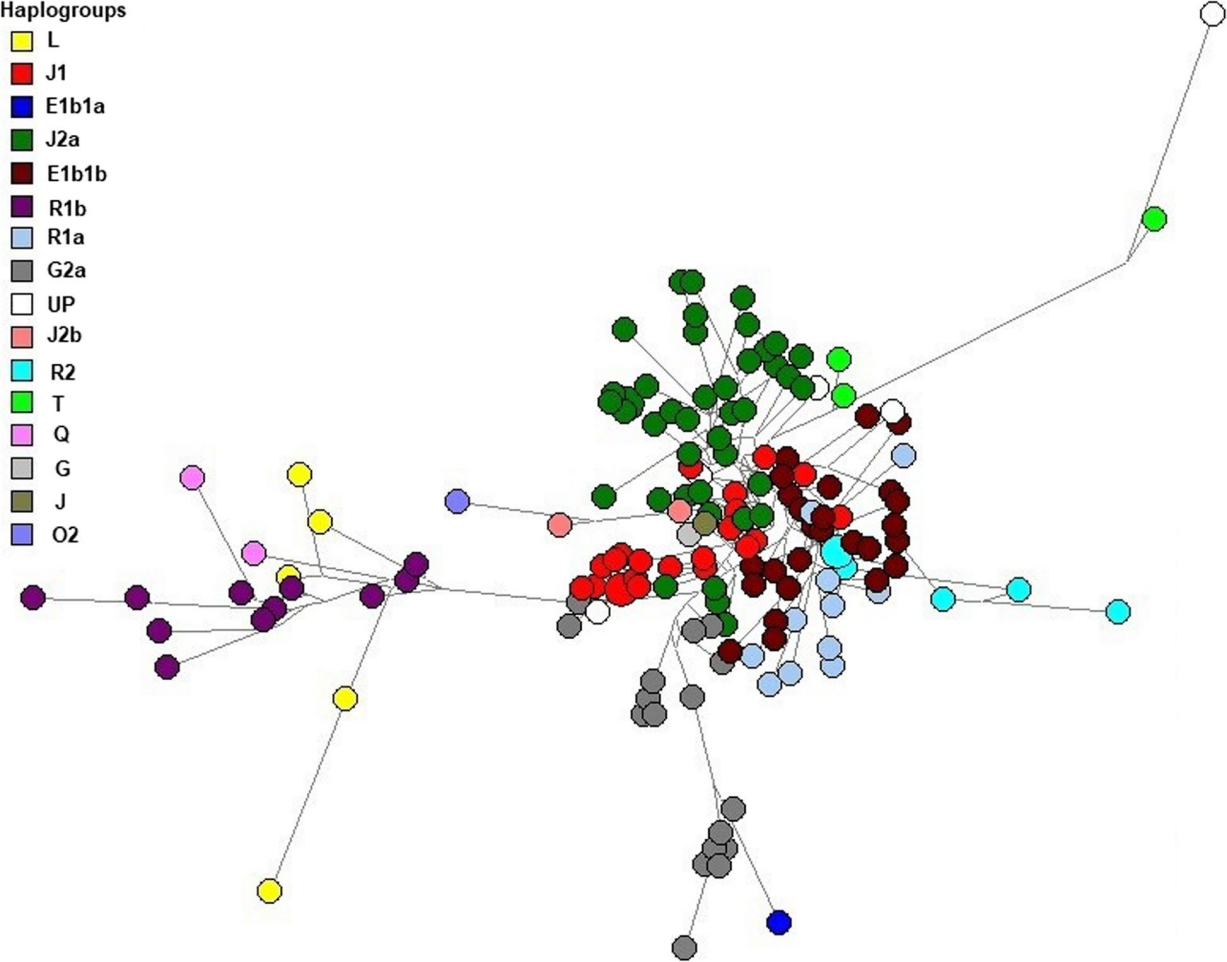
Median-joining network for the haplotypes of 157 Sorani Kurds, constructed from data on 17 Y-STRs. Circles represent haplotypes, with the area proportional to sample size, and lines between them proportional to the number of mutational steps. The colours representing the haplogroups are indicated in the left key. UP = unpredicted

Median-joining Y-STR network was calculated for the Sorani Kurd haplotypes using NETWORK v5.0.1.0 and edited by NETWORK publisher v2.1.1.2 (Fluxus Technology Ltd.). The median-joining calculation was based on Whit Athey’s haplogroup prediction results and six major clusters were obtained from the haplotypes of the present study: J2a, E1b1b, J1, G2a, R1a and R1b (Fig. 3).

### Population comparison

**Fig. 4:**
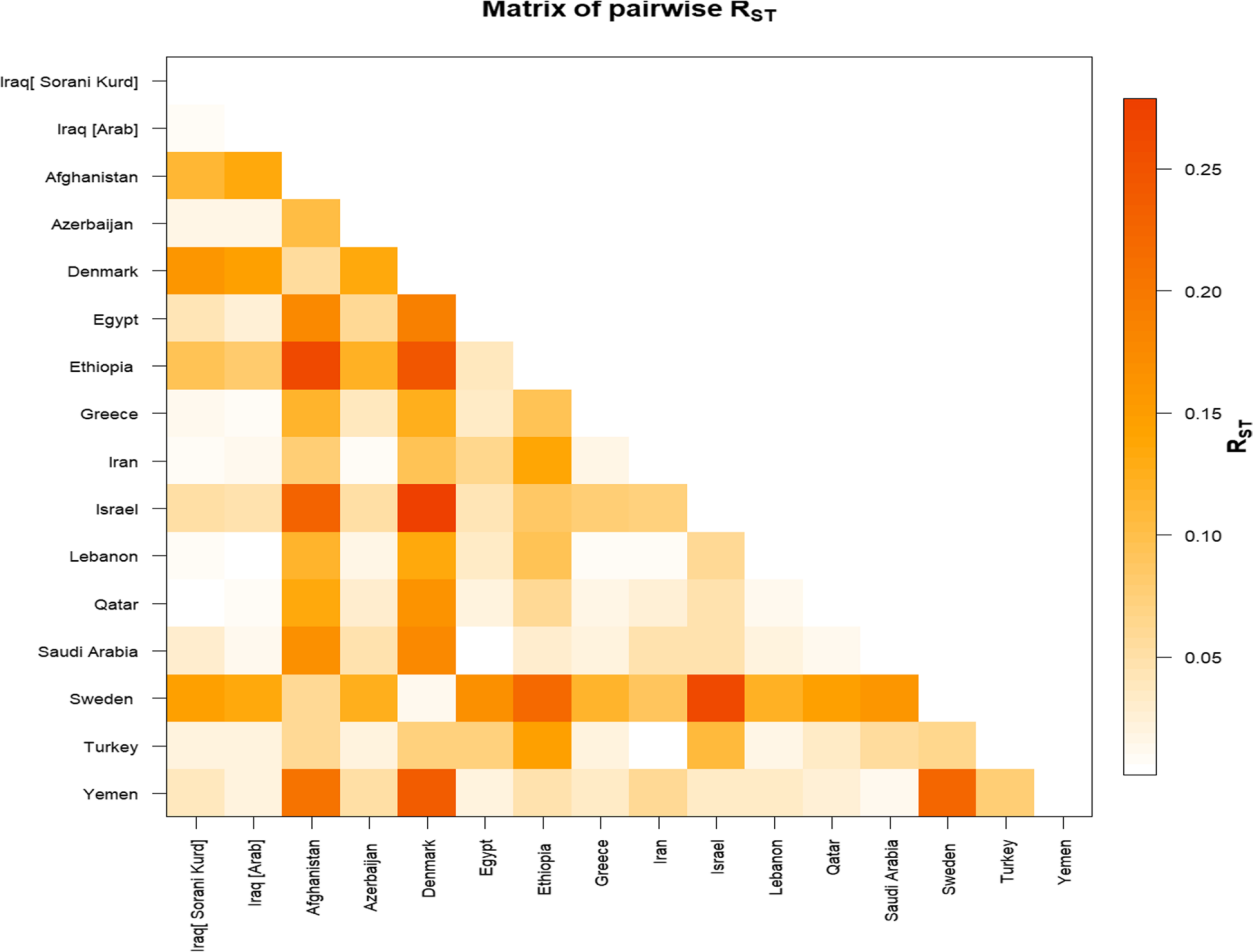
The matrix of pairwise genetic distance based on R_st_ of Y-STRs. The Sorani Kurd was closest to Qatar, Lebanon, Iraqi Arabs and Iran and furthest from Denmark, Sweden, Afghanistan and Ethiopia

The haplotypes of the Sorani Kurd were compared to fifteen other population haplotypes using YHRD databases: Iraqi (Arabs), Iran, Qatar, Saudi Arabia, Lebanon, Yemen, Israel, Turkey, Afghanistan, Azerbaijan, Egypt, Ethiopia, Greece, Denmark and Sweden. The population pairwise genetic distances (R_st_) were calculated between the studied population and the previously reported populations (Table S5); the results are shown in Fig. 4. A multidimensional scaling (MDS) plot was performed from R_st_ distances (Fig. S4), and the result showed that the Sorani Kurdish population clustered with Qatar, Lebanon, Iraq (Arab), Iran, Greece, Azerbaijan and Turkey. The closest populations to the Sorani Kurds were Qatar (R_st_ = 0.0042), Lebanon (R_st_ = 0.0078), Iraqi Arabs (R_st_ = 0.008) and Iran (R_st_ = 0.0084). The furthest populations were Denmark (R_st_ = 0.1575), Sweden (R_st_ = 0.1467), Afghanistan (R_st_ = 0.1136) and Ethiopia (R_st_ = 0.0951).

**Fig. 5:**
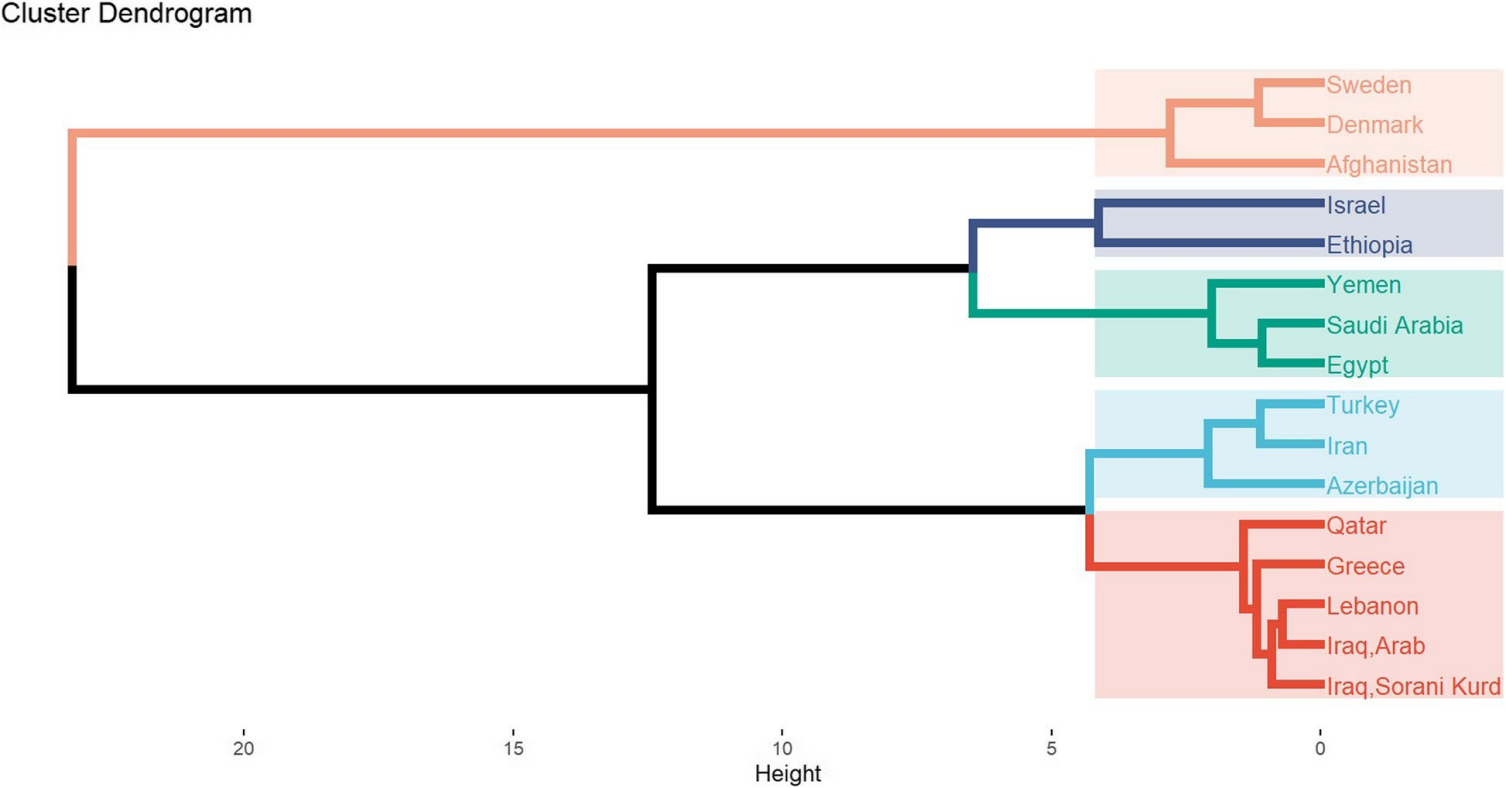
The dendrogram shows five clusters of the 15 populations using R statistical software (version 4.0.3). This dendrogram was generated based on the R_st_ genetic distance values

Population clustering was performed based on R_st_ values generated by the YHRD tools. The R statistical software was used to calculate and generate the hierarchical relationships among the 15 different populations [23]. Five clusters were obtained (K = 5) as shown in Fig. 5. Iraqi Sorani Kurds, Iraqi Arabs, Lebanon, Greece and Qatar fell into one cluster. The rest of the western Asia populations were divided among three clusters: Iran, Turkey and Azerbaijan fell into one cluster, Yemen and Saudi Arabia into another with Egypt, while Israel was clustered with Ethiopia. The last group contained Afghanistan clustered with the European populations, Sweden and Denmark.

**Fig. 6:**
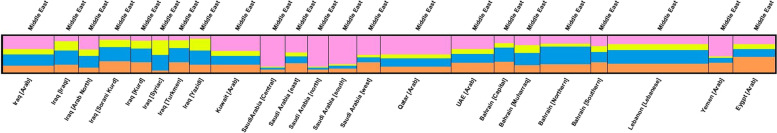
The HapMap of the Y-STR haplotypes using 17 STR markers. The results showing 4 clusters using 23 populations (3833 individuals)

The HapMap of the Y-STR with the middle eastern populations was generated. The 23 populations utilised in the structure analysis have their Y STR data acquired from published data: Iraq [18, 19, 24] and this study, Kuwait [25], Saudi Arabia [26], Qatar (YHRD accession YC000494), Yemen (YHRD accession YA005529), Lebanon [24], UAE [27], Bahrain [28] and Egypt [29]. Within each population, the Y-HapMap STRs revealed a more distinct sub-grouping of countries (Fig. 6).

## Discussion

Kurds are divided geographically, linguistically and tribally as a consequence of earlier invasions and migrations. Kurdish tribes are found throughout Iran, Turkey, Syria and Iraq and many of the tribes in Iraq live in and around Sulaymaniyah province in Iraqi Kurdistan [30, 31]. Therefore, the study of population genetics in the ethnic Kurd was necessary to trace the paternal and maternal lineage of the Kurdish tribes.

In the present study, the Y-STRs were used to determine the haplotype frequency and genetic variation of 17 loci among Sorani Kurds in Sulaymaniyah province. The results revealed that the highest genetic diversities were for DYS385a/b (0.848) and DYS458 (0.828) loci. The lowest genetic diversity for the Sorani Kurdish population was DYS392 (0.406). These results are similar to those previously reported for the Iraqi Arab population and Kurdish people in northern Iraq [12, 19, 20].

STR duplicates were confirmed in two individuals at two loci, DYS19 and Y GATA H4. A double allele at locus DYS19 was observed in one sample. Double alleles at the same locus were previously observed in the Iraqi population with the same duplicated alleles (15, 16) [18]. Other studies observed double alleles in DYS19 [24, 32, 33]. In the YHRD Release 66, the mutation rate of the DYS19 locus was 2.12e-3 (42 in 19,807) and this duplication (15, 16) was at a frequency of 0.051%. A duplication of the YGATA H4 locus was observed in one individual with the values 11, 12. A global study of the Y chromosomal haplotypes showed that one sample carried duplicated alleles at YGATA H4 with the values of 11, 12 [24]. The YHRD (Release 66) contained a total of four observations (0.0013%) of the YGATA 11, 12 duplication. In addition, null alleles were found in the Sorani Kurdish population in two loci, DYS448 and YGATA H4. These null alleles are most likely due to deletions of the target region or primer binding site regions or by mutations in the primer binding sites [34]. The previous study of the Y chromosomal haplotypes showed that the DYS448 has the highest level of null alleles in 51 countries (59/19,630) [24]. Null alleles at various loci were observed in the Indian population, including the locus DYS448 [24]. Based on the YHRD (Release 66), the mutation rate of the DYS448 locus was 1.37e-03 (15 in 10,935) and 827 null alleles were observed; whereas the mutation rate of the YGATA H4 was 2.51e-03 (30 in 11,970) and observations of 22 null alleles were reported.

In this study, off-ladder alleles were observed at locus DYS635 allele 27 (three samples) and locus Y GATA H4 allele 14 (two samples). However, these off-ladder alleles of the Yfiler kit are present in the allelic ladder of other commercial kits such as a Yfiler™ Plus and PowerPlex® Y23 System [35, 36]. This addition in the other commercial kits can be helpful in appropriately designating rare alleles.

Y-STR haplogroups were inferred using Whit Athey’s Haplogroup predictor. The samples of the Sorani Kurds were classified into 18 different haplogroups. The major ones (> 10%) were J (42.67%), R (18.47%), E (17.19%) and G (10.83%). The subclades of these haplogroups were J2 (28%), E1 (17.19%), R1 (14.64%), J1 (14%), G2 (10.83%) and R2 (3.8%). The results of the present study are in agreement with previous results that the most common haplogroups in the Kurdish population are J and R. Previous studies concluded that the haplogroup J is a common male lineage in West Asia [37]. However, the phylogeography of this haplogroup is complex. The two sub-haplogroups J1 and J2 are similar in distribution, but J2 is most common among modern Kurdish [19], Jewish [38], and Iranian [9] and is also found in tribal populations inhabited in different parts of India [39]. While the maximal frequency of the subclade J1 is in Arab-speaking populations. Predictions indicated the predominance of haplogroup J1 in Iraqi Arabs (36.6%) [18], in Saudi Arabia (71%) [26], and in Kuwait (37%) [40], whereas haplogroup prediction in the Bahraini population suggested that the most common subclade is J2 (27.6%) followed by J1 (23%) [28].

Members of the haplogroup R are widespread in Europe, R1a being most common in eastern Europe and R1b in western Europe [41, 42]. Studies indicated that the haplogroup R originated in north Asia about 27,000 years ago and is widespread in western, central and southern Asia [42, 43]. In addition, haplogroup R is one of the largest clades in the Indian subcontinent [42]. Members of the clade R are also found at high frequencies in the central-western part of the African continent [44]. In the present study, the second major haplogroup among the Sorani Kurds was R 18.4% (29/157) (R1a = 12, R1b = 11, R2 = 6). The previous results on the Kurdish population in northern Iraq revealed that the major sub-haplogroup was R1a 17.17% (17/104); four other samples belonged to R1b sub-haplogroup (4.04%) while R2 was not observed [19].

Genetic studies indicated that the highest frequency of the haplogroup E1b1b-M35 is in north Africa and reaches an average frequency of 42–45% across the region [15, 45]. In the current study, high frequencies of the E1b1b-M35 sub-haplogroup were observed 16.56% (26/157) while the E1b1a sub-haplogroup was found in only one individual (0.64%).

The haplogroup G is most common in the Caucasus, Near/Middle East and in southern Europe. Archaeological research estimated the origin of the clade G, adjacent to eastern Anatolia. The haplogroup G, with the haplogroup J2, has been associated with the spread of agriculture into Europe [46]. In the present study, the haplogroup G, particularly sub-haplogroup G2a-P15, was frequently observed 10.83% (17/157) in the Sorani Kurdish population. Observations of the sub-haplogroups E1b1b-M35 and G2a-P15 were also significant from the earlier study on the paternal lineage of Northern Iraqi Kurds, E1b1b-M35 (13/104) (12.5%) and G2a-P15 (8/104) (7.69%) [19].

However, these slight differences in the genetic parameters were expected. The current study, the first to our knowledge, focuses on one group of the Kurdish population (Sorani Kurds) in Iraq, separated from the Kurmanji Kurdish group in the northwest of Iraq on the border with Turkey. In addition, a higher number of population samples were collected than in the previous paternal lineage studies of the Kurdish population [19, 20, 47], which is also an important consideration in obtaining more reliable results with greater precision and power.

The Sorani Kurdish population was compared with 15 other populations in the YHRD database. A pairwise population genetic distance (R_st_) revealed significant differences between Sorani Kurds and populations from western Europe and Africa, while similarities were observed with the west and central Asian countries. However, genetic distance results are strongly influenced by the loci number and sample sizes. Increasing the loci numbers will improve the precision estimates of the genetic differences. In addition, larger sample sizes per population can provide more accurate mean values if insufficient loci are available [48].

The Y-STR HapMap developed in this study revealed not only closer geographical proximity of the population samples, but also a more distinct sub-grouping of the respective populations. The results of the present study show that the Sorani Kurdish population is part of the Middle Eastern population.

## Conclusions

Human Y-STR markers provide powerful results for haplotype analysis and haplogroup prediction, which lead to individual geographical origins. In this study, a database of 17 Y-STR loci for the Sorani Kurdish population was established. The highest and lowest gene diversities were found at the loci DYS385a/b and DYS392, respectively, and the J2 haplogroup predominated. The findings also show that the Sorani Kurdish population is genetically more similar to populations in Western and Central Asia than to populations in Europe and Africa. Because genetic data on the diversity of the Kurdish population is limited, providing Sorani Kurd paternal lineage data may aid in developing a better understanding of this Kurdish ethnic group’s bio-geographic ancestry.

## Methods

### Sampling and DNA extraction

Blood samples were collected with written informed consent from 157 Sorani Kurd males aged 18 years old and above, using heparin or EDTA tubes. DNA was extracted using a Prime Prep DNA isolation kit (GeNet Bio-Korea) according to the manufacturer’s protocol. The purity and concentration of the DNA were determined by using an Eppendorf Biophotometer Plus (Eppendorf-Germany).

### Polymerase chain reaction (PCR)

The AmpFLSTR™ Yfiler™” PCR Amplification Kit contains 17 loci: DYS438, DYS393, DYS385a/b, DYS389I/II, DYS458, DYS437, DYS391, DYS392, DYS635 (Y GATA C4), Y GATA H4, DYS19, DYS390, DYS439, DYS456 and DYS448. Simultaneous amplification was conducted following the manufacturer’s instructions (Thermo Fisher Scientific). The amplified fragments were separated on an ABI Prism® 310 Genetic Analyzer, and allele calling was performed with GeneMapper® V.4.1. ID software (Thermo Fisher Scientific).

### Data analysis

The haplotype frequencies were calculated using GenAIEx 6.5 [49]. The forensics statistics including gene diversity (GD), polymorphism information content (PIC), match probability (PM) and power of discrimination (PD) were calculated using the STRAF online tool [50].

Haplogroup predictions from Y-STR values of the Sorani Kurdish population were inferred using Athey’s Haplogroup Predictor [51]. Median-joining networks were constructed by the software NETWORK v5.0.1.0 and NETWORK Publisher v2.1.2.5 (Fluxus Technology Ltd.) following the author’s recommendations [52, 53].

Population comparisons were performed using the YHRD database (https://yhrd.org (accessed on 17/01/2022)) [54]. The online AMOVA program was used to calculate the R_st_ among the populations and the multidimensional scaling (MDS) was performed based on Kruskal’s non-metric MDS algorithm [55].

Population structure in the Arabian Peninsula was investigated using the program STRUCTURE version 2.3.7 with an admixture model [56]. The HapMap was generated for the Y-STR data for 17 markers of 23 populations (3833 individuals).

## Supplementary Information

**Additional file 1: Table S1.** Y-STR haplotypes and predicted haplogroups for 157 Iraqi [Sorani Kurd] males. **Table S2.** Allele frequencies and sample size by locus of the 17-STR loci for the 157 males of the Sorani Kurd. **Table S3.** Gene diversity (GD), polymorphism information content (PIC), match probability (PM) and power of discrimination (PD) of the Sorani Kurd males. **Table S4.** Haplogroup distribution in Sorani Kurdish population. **Table S5.** Sum of squared size difference (R_st_) in 15 populations.

**Additional file 2: Fig. S1.** Chart showing match probability and gene diversity for each of the 17 loci in the Sorani Kurdish population. **Fig. S2.** Variant alleles, duplications and deletions, at different loci in four different individuals. **Fig. S3.** Chart showing Y-haplogroup distribution in the Sorani Kurdish population. **Fig. S4.** Multidimensional scaling plots of fifteen different populations based on R_st_ values.

## Data Availability

The data generated in this study have been deposited with links to BioProject accession number PRJNA868298 in the NCBI BioProject database (https://www.ncbi.nlm.nih.gov/bioproject/). Haplotype data has been uploaded to the YHRD: Contribution YA005683, release R66. Supplementary data associated with this article can also be found in the supplementary materials.

## Abbreviations

STR: Short tandem repeat
DNA: Deoxyribonucleic acid
BC: Before Christ
PCR: Polymerase chain reaction
GD: Gene diversity
YHRD: Y-chromosome STR haplotype reference database
EDTA: Ethylenediamine tetraacetic acid
Rst: A pairwise population genetic distance
HapMap: Haplotype map
PIC: Polymorphism information content
PM: Match probability
PD: Power of discrimination
GenAIEx: Genetic analysis in Excel
STRAF: STR analysis for forensics
MDS: Multidimensional scaling
AMOVA: Analysis of molecular variance
