# Supplementary material for: Population genetic study of 17 Y-STR Loci of the Sorani Kurds in the Province of Sulaymaniyah, Iraq
Source: BMC Genomics. 2022 Nov 21;23:763. doi: 10.1186/s12864-022-09005-6 (PMC9682651; doi:10.1186/s12864-022-09005-6)
Supplement: Supplementary file 2 — Additional file 2: Fig. S1. Chart showing match probability and gene diversity for each of the 17 loci in the Sorani Kurdish population. Fig. S2. Variant alleles, duplications and deletions, at different loci in four different individuals. Fig. S3. Chart showing Y-haplogroup distribution in the Sorani Kurdish population. Fig. S4. Multidimensional scaling plots of fifteen different populations based on Rst values. [file 12864_2022_9005_MOESM2_ESM.docx]

**Figure Legends**

**Figure S1:** Chart showing match probability and gene diversity for the 17 loci of the Sorani Kurds.

**Figure S2:** Variant alleles, duplications and deletions, at different loci in four different individuals.

**Figure S3:** Chart showing Y-haplogroup distribution in the Sorani Kurdish population.

**Figure S4:** Multidimensional scaling plots of fifteen different populations based on Rst values.

**Supplementary figures**


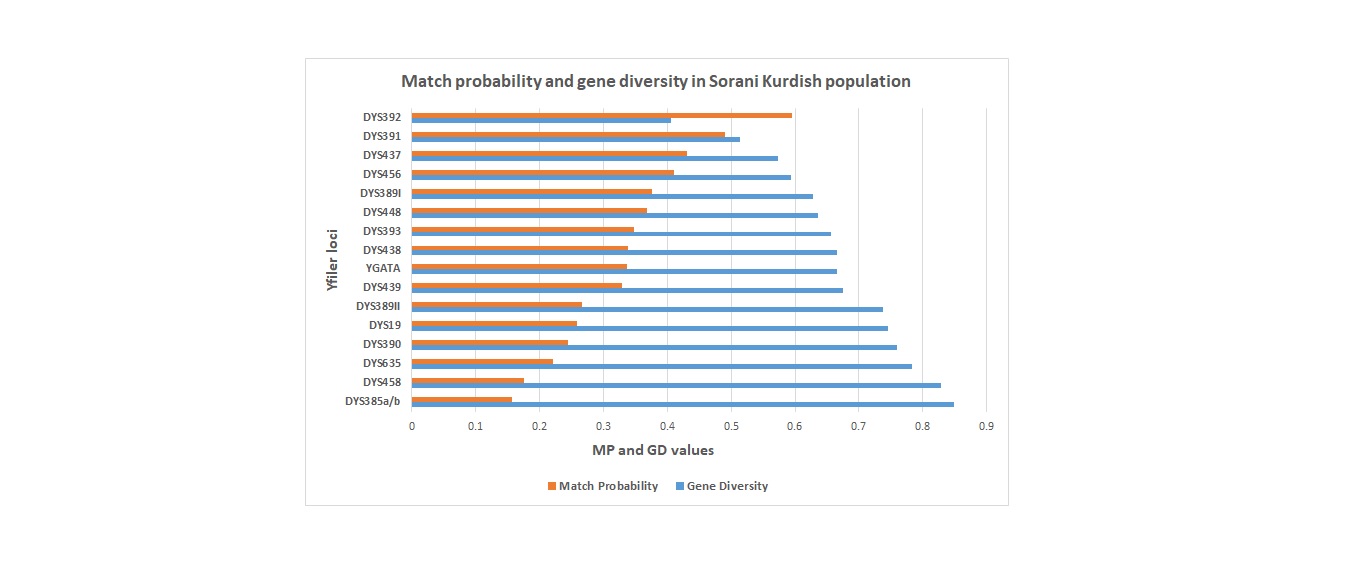


**Figure 1**


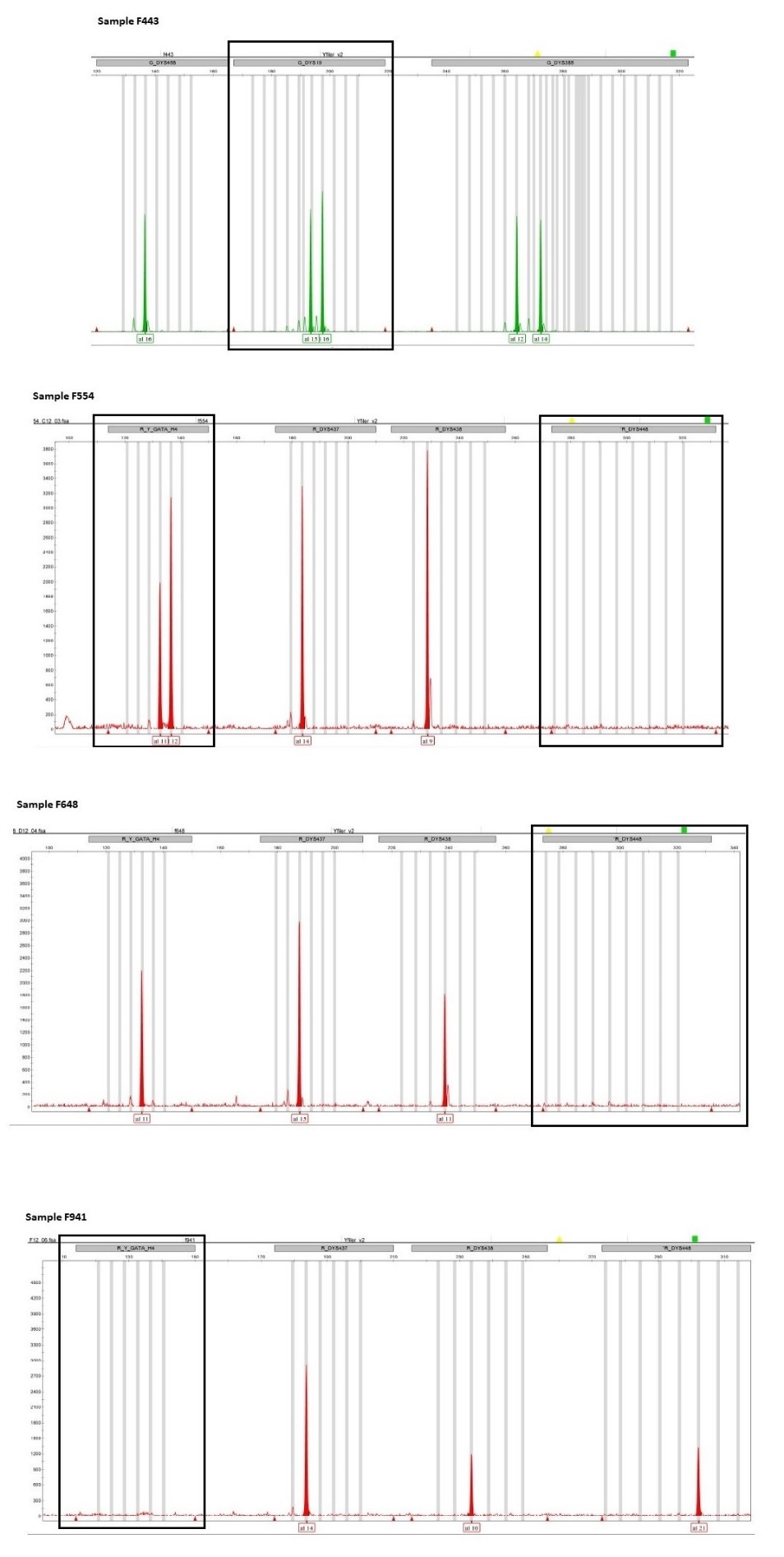


**Figure 2**


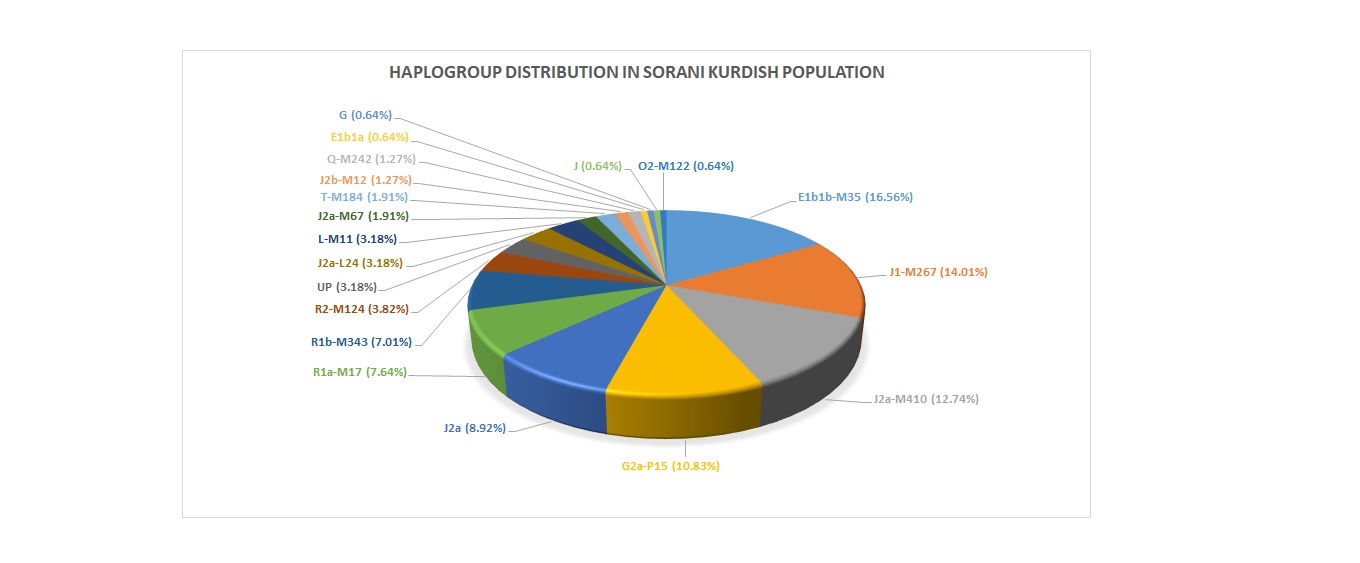


**Figure 3**


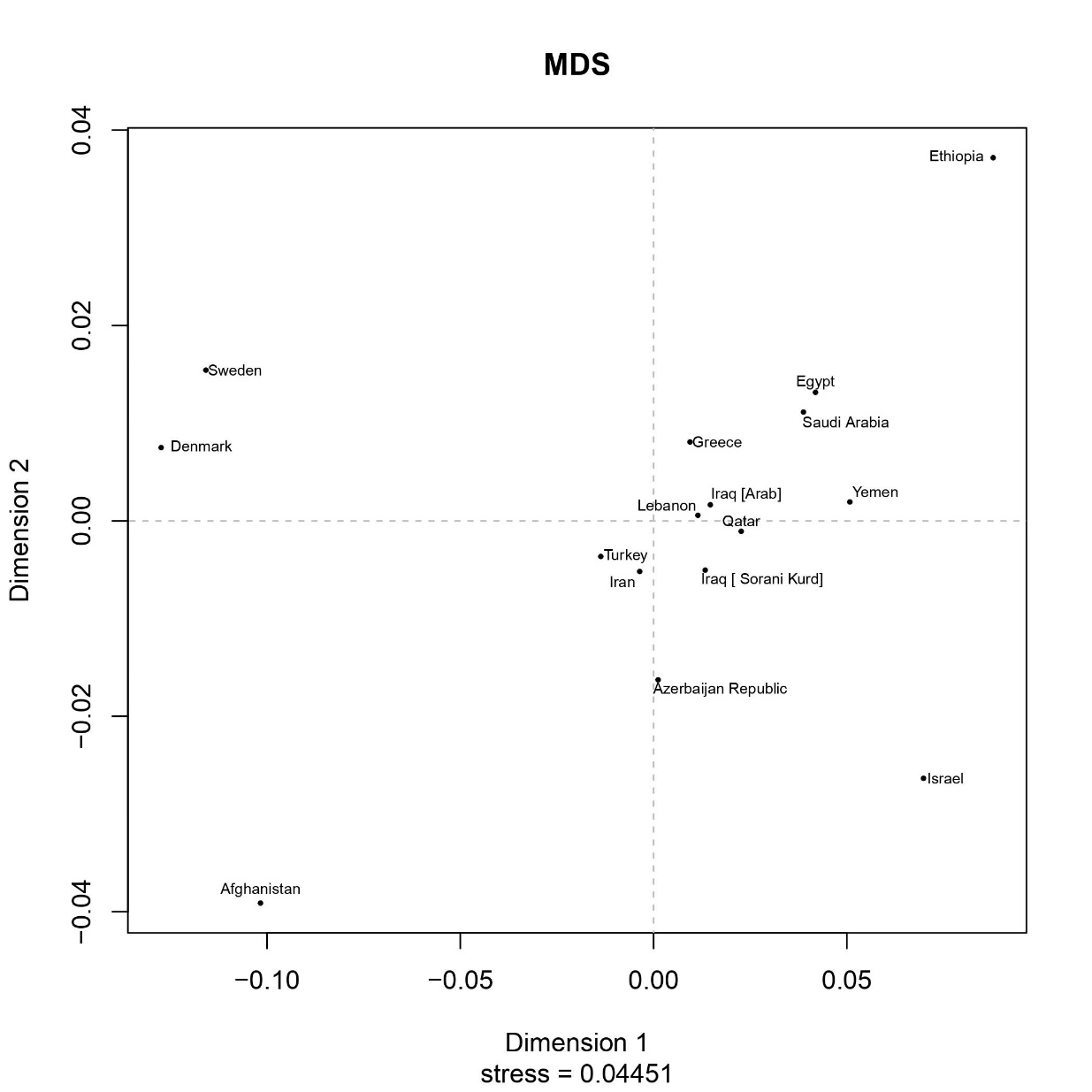


**Figure 4**
